# Novel glucose-lowering drugs and the risk of acute kidney injury in routine care; the Stockholm CREAtinine Measurements (SCREAM) project

**DOI:** 10.1007/s40620-022-01505-8

**Published:** 2022-12-02

**Authors:** Jim Alkas, Alessandro Bosi, Arvid Sjölander, Peter Barany, Carl-Gustaf Elinder, Edouard L. Fu, Juan Jesus Carrero

**Affiliations:** 1 Department of Medical Epidemiology and Biostatistics, Karolinska Institutet, Stockholm, Sweden; 2 Department of Clinical Science, Intervention and Technology, Karolinska Institutet, Stockholm, Sweden; 3 Division of Pharmacoepidemiology and Pharmacoeconomics, Department of Medicine, Brigham and Women’s Hospital and Harvard Medical School, Boston, USA; 4 Department of Clinical Sciences, Karolinska Institutet, Danderyd Hospital, Stockholm, Sweden

**Keywords:** SCREAM, AKI, Creatinine, Diabetes, Glycemic control

## Abstract

**Introduction:**

Little is known about the comparative effects of sodium glucose cotransporter-2 inhibitors (SGLT2i), glucagon-like peptide-1 receptor agonists (GLP1-RA), or dipeptidyl peptidase-4 inhibitors (DPP-4i) on the risk of acute kidney injury (AKI) in routine care, which may differ from the controlled setting of trials.

**Methods:**

Observational study comparing risks of AKI among new users of SGLT2i, GLP1-RA or DPP-4i in the region of Stockholm, Sweden, during 2008–2018. AKI was defined by ICD-10 codes and creatinine-based KDIGO criteria. We used inverse probability of treatment weighting (IPTW) to adjust for 60 potential confounders, weighted Kaplan–Meier curves and Cox regression to estimate hazard ratios and absolute risks.

**Results:**

We included 17,407 participants who newly initiated DPP-4i (*N* = 10,605), GLP1-RA (*N* = 4448) or SGLT2i (*N* = 2354). Mean age was 63 years (39% women) and median (IQR) eGFR was 89 (73–100) ml/min/1.73 m^2^. During a median follow-up of 2.5 years, 1411 participants experienced AKI. SGLT2i users had the lowest incidence rate of AKI, 18.3 [CI 95% 14.1–23.4] per 1000 person years, followed by GLP1-RA (22.5; 19.9–25.3) and DPP-4i (26.6; 25–28.2). The weighted 3-year absolute risk for AKI was 5.79% [3.63–8.52] in the SGLT2i group, compared with 7.03% [5.69–8.69] and 7.00% [6.43–7.58] in the GLP1-RA and DPP-4i groups, respectively. The adjusted hazard ratio was 0.73 [CI 95% 0.45–1.16] for SGLT2i vs. DPP-4i, and 0.98 [CI 95% 0.82–1.18] for GLP1-RA vs. DPP-4i.

**Conclusion:**

This study of routine care patients initiating novel glucose-lowering drugs showed similar occurrence of AKI between therapies, and suggests lower risk for SGLT2i.

**Supplementary Information:**

The online version contains supplementary material available at 10.1007/s40620-022-01505-8.

## Introduction

Diabetes mellitus type 2 (T2DM) is a global health problem with a high risk of complications and death [1]. Sodium glucose cotransporter-2 inhibitors (SGLT2i) are novel oral diabetes medications that, in addition to glucose lowering effects, have been shown in clinical trials to reduce the risk of hospitalization for heart failure, kidney disease progression, kidney failure, death from renal or cardiovascular causes, and all-cause mortality [2–7]. On the basis of these trials, clinical guidelines recommend administering SGLT2i in addition to metformin to patients with or at high risk for atherosclerotic cardiovascular disease, those with clinical heart failure, and those with chronic kidney disease (CKD) [8, 9].

There is often an acute drop in estimated glomerular filtration rate (eGFR) during the first weeks of SGLT2i therapy, [4] which may lead to acute kidney injury (AKI) [10]. A review of more than 100 spontaneous reports of confirmable cases of AKI related to SGLT2i led the US Federal Drug Administration (FDA) to issue a warning in December 2015 stating that SGLT2 inhibitors might cause AKI [11]. The FDA recommended following and quantifying this risk in post-marketing surveillance studies [10, 11].

**Fig. 1:**
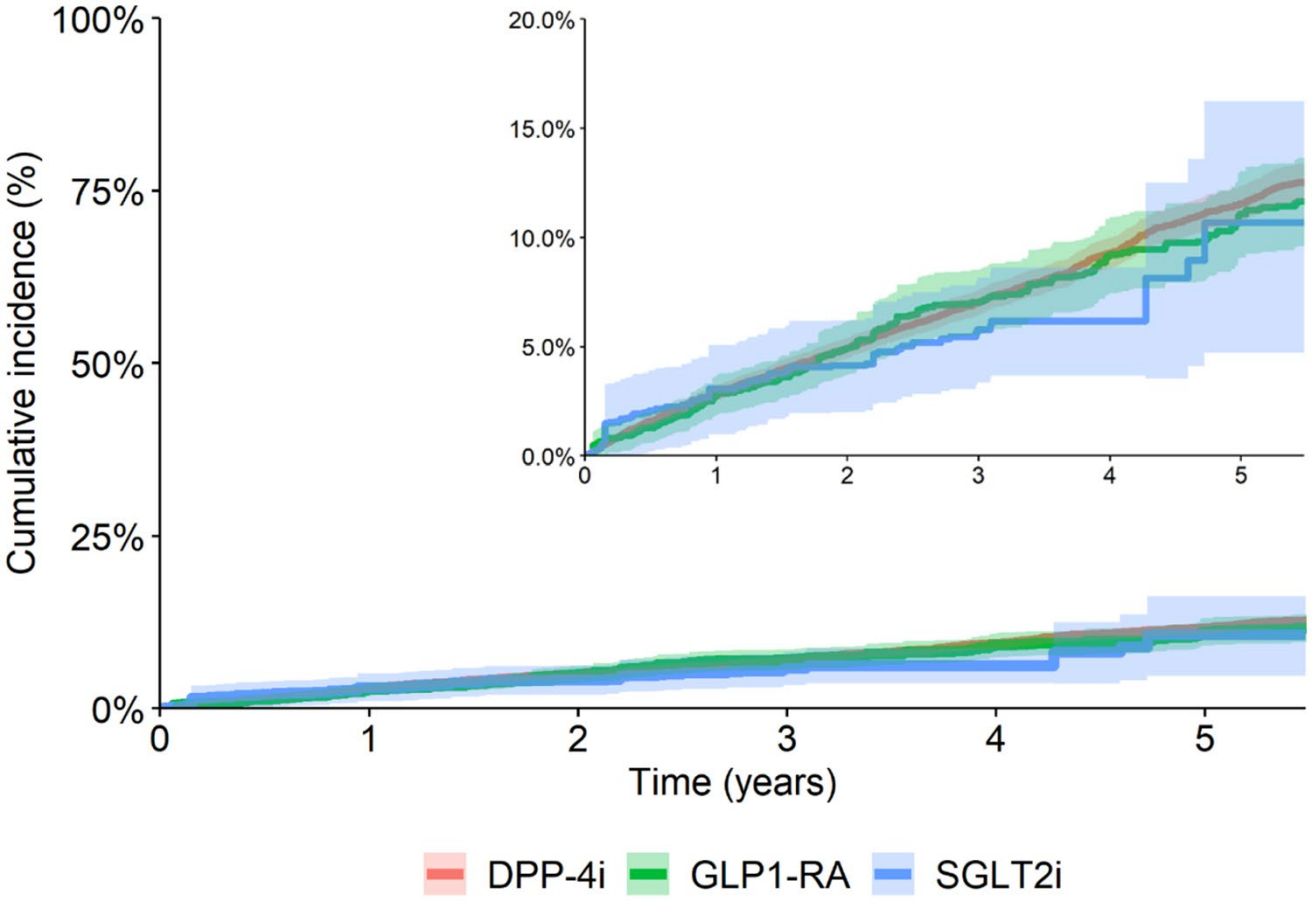
Cumulative incidence curve, overall and with the Y axis magnified. Kaplan–Meier plots for weighted cumulative incidence of acute kidney injury (AKI) stratified by DPP-4i, GLP1-RA or SGLT2i treatment. Light-colored areas represent 95% confidence intervals. *X* axis truncated at 5.5 years. *DPP-4i* dipeptidyl peptidase-4 inhibitor, *GLP1-RA* glucagon-like peptide-1 receptor agonist, *SGLT2i* sodium-glucose cotransporter-2 inhibitor (color figure online)

Case reports contrast with trial data: a recent network meta-analysis of RCTs indicates that SGLT2 inhibitors may have a lower AKI risk compared with dipeptidyl peptidase 4 (DPP-4) inhibitors and glucagon-like peptide 1 receptor agonists (GLP-1RAs) [12]. However, in these trials, AKI was documented as an adverse event rather than a prespecified outcome, and the strict inclusion/exclusion criteria alongside stringent monitoring protocols may have led to an underestimation of adverse event rates. In the heterogeneous setting of routine clinical practice, various North American studies have reported similar AKI event rates among new users of SGLT2i compared to other oral diabetes medications [13–15]. However, the family of oral diabetes medications is heterogeneous, and risks may be better ascertained through comparative safety analyses. Three studies have compared AKI risks of SGLT2i versus DPP-4 inhibitors and GLP-1RAs, observing either no difference between therapies, [16] or a lower AKI risk for SGLT2i [17, 18]. Identified limitations for these studies include the use of administrative codes to identify AKI, which are insensitive, lack information on baseline eGFR, which may modulate subsequent AKI risks, and restrictions by certain health insurance companies, or to persons older than 65 years. Replication of consistent adverse drug event relationships across geographically distinct health systems and variations in prescribing practices may provide greater confidence in results. Against this background, we conducted an observational study comparing risks of AKI among routine care users of novel glucose-lowering drugs in Stockholm (Fig. 1).

## Materials and methods

### Data source

The study population was derived from the Stockholm CREAtinine Measurements (SCREAM) project, a health care utilization cohort that includes all residents of the region of Stockholm, Sweden, between 2006–2019 [19]. In 2019, the region had a population of approximately 2.3 million citizens, to whom a single unified health system provides universal health care access. Using the unique personal identification number of each Swedish resident, administrative databases containing extensive information on demographics, health care utilization and comorbidities, laboratory test results and drug dispensation records were linked, thereby providing comprehensive structured health records.

### Study design

We included any adult (age ≥ 18 years old) residing in Stockholm with a clinical diagnosis of diabetes, who newly initiated SGLT2i, GLP1-RA or DPP-4i therapy between 2008-01-01 and 2018-12-31. We established that the patient should have had at least one outpatient serum/plasma creatinine test in the year prior to therapy start to calculate his/her kidney function (Supplemental Figure S1). The date of treatment initiation was defined as the index date and start of follow-up. Then, we excluded patients with type 1 or gestational diabetes mellitus, as well as those with previous history of AKI (by issued clinical diagnosis), with eGFR < 15 ml/min/1.73 m^2^, on dialysis treatment or that had a history of kidney transplantation.

### Exposure and covariates

The study exposure was new initiation of SGLT2i, GLP1-RA or DPP-4i therapy, defined as the first dispense during the study period with an absence of any dispensation of the drugs of interest over the previous 12 months. We did not consider treatment switches during follow-up, if any, in the analysis. We extracted information at index date for 60 covariates including demographics (age and sex, year of therapy start and highest attained level of education), laboratory tests (eGFR and HbA1c), history of comorbidities, ongoing medications, and recent healthcare utilization. eGFR was calculated using routine ambulatory isotope-dilution-mass-spectrometry–traceable plasma creatinine measurements and applying the 2009 CKD-EPI equation without correction for race [20]. eGFR at baseline was defined as the average of all outpatient creatinine measurements performed in the preceding 12 months and categorized as per KDIGO criteria in stages of G severity [21]. To capture healthcare utilization and disease severity, we also considered the number of overall and disease-specific outpatient/inpatient encounters in the 12 months prior to the index date. Defining algorithms are detailed in Supplemental Table S1.

### Study outcome

The study outcome was the occurrence of AKI during follow-up. AKI was identified by a combination of diagnoses (ICD-10 code N17) in outpatient or hospital care, the need for acute renal replacement therapy, and transient creatinine elevations during hospitalization according to KDIGO criteria [22] (increase in creatinine ≥ 26 μmol/l over 48 h or ≥ 1.5 times within 7 days). We defined the “baseline” creatinine as the mean of the last outpatient creatinine measurements 7–365 days prior to hospital admission. Definitions are detailed in Supplemental Table S2. Patients were followed until occurrence of AKI, death, emigration from the region or administrative censoring on 2018–12-31. There was no loss to follow-up.

### Statistical analyses

Categorical variables were reported as frequencies and percentages. Continuous variables were reported as median with interquartile range (IQR). There were no missing data in any of the covariates assessed, except for attained level of education, which was missing in < 3% of participants. Due to the low amount of missingness, we treated “missingness” as a separate category for this variable in the analysis.

We used inverse probability of treatment weighting (IPTW) to control for baseline confounding [23]. We estimated the probability of receiving GLP1-RA or SGLT2i versus DPP-4i (reference) as a function of the baseline covariates listed above using a multivariable logistic regression model. Weighting was considered appropriate if the standardized mean difference (SMD) between treatment groups was < 0.1. Weights were stabilized to increase precision by adding the marginal probability of treatment to the numerator of the weights.

We first estimated the total number of events and incidence rates for each group without weighting. We then used weighted Cox regression to estimate hazard ratios (HR) and 95% confidence intervals (95% CI) between treatment groups, with time since initiation as the underlying time scale. In order to compare outcome rates between SGLT2i and GLP1-RA, we additionally repeated the weighting with GLP1-RA as the reference. Covariates that did not achieve balance after IPTW were controlled for by adding them as covariates to the model.

Weighted Kaplan–Meier curves were plotted to display the cumulative incidence of outcomes over the follow-up period for each medication. We then obtained weighted absolute risks and risk differences and used a bootstrap resampling technique to construct 95% confidence intervals, using 1000 samples [24]. As a sensitivity analysis, we tested the robustness of IPTW weighting by comparing against an alternative method of overlapping weights [25]. All the analyses were performed using R statistical software.

## Results

We included 17,407 participants who met the inclusion and exclusion criteria (Figure S1). Of these, 10,605 initiated DPP-4i treatment (61%), 4448 initiated GLP1-RA (26%) and 2354 initiated SGLT2i (13%). Overall, mean age was 62.8 years, 61% were male and median (IQR) eGFR was 89 (73–100) ml/min/1.73m^2^. Unweighted characteristics by treatment group are shown in Supplemental Table S3. In brief, DPP-4i users were generally older and with a slightly lower median eGFR level than participants who started on GLP1-RA or SGLT2i. Users of SGLT2i (of whom 59% started on empagliflozin, 40.5% on dapagliflozin, and < 1% on canagliflozin) included a higher proportion of men and with cardiovascular comorbidities, mainly history of ischemic events. There were no major differences in baseline HbA1c between groups, but patients on GLP1-RA tended to have a higher proportion of concomitant insulin treatment. After weighting, most covariates achieved balance (shown in Table 1 with additional covariates listed in Tables S3 and S4), except for calendar year and CKD stage.

**Table 1:** Selected baseline characteristics of DPP-4i, GLP1-RA and SGLT2i after propensity score weighting

	DPP-4i	GLP1-RA	SGLT2i	SMD DPP-4i (Ref.)	SMD GLP1-RA (Ref.)
Number of individuals	17,573	17,082	13,645		
Age, median, [IQR]	63 [54, 72]	63 [55, 71]	64 [56, 72]	0.08	0.07
Women	6784 (39%)	6408 (38%)	5384 (39%)	0.03	0.03
eGFR, median [IQR] (ml/min/1.73 m^2^)	89 [72, 100]	90 [74, 100]	87 [69, 99]	0.08	0.07
CKD stage				0.14	0.13
G1 (≥ 90 ml/min/1.73 m^2^)	8885 (51%)	8787 (51%)	6277 (46%)		
G2 (60–89 ml/min/1.73 m^2^)	6396 (36%)	6162 (36%)	5104 (37%)		
G3a (45–59 ml/min/1.73 m^2^)	1416 (8%)	1201 (7%)	1293 (9%)		
G3b (30–44 ml/min/1.73 m^2^)	754 (4%)	803 (5%)	971 (7%)		
G4 (15–29 ml/min/1.73 m^2^)	123 (1%)	128 (1%)	0 (0%)		
Hba1c, median [IQR] (mmol/mol)	61 [54, 72]	62 [54, 74]	61 [52, 72]	0.04	0.04
Education				0.07	0.07
Compulsory school	4400 (25%)	4290 (25%)	3676 (27%)		
Secondary school	7647 (44%)	7413 (43%)	5553 (41%)		
University	5160 (29%)	4910 (29%)	4188 (31%)		
Missing	366 (2%)	468 (3%)	228 (2%)		
Acute coronary syndrome	1737 (10%)	1722 (10%)	1335 (10%)	0.01	0.01
Other ischemic heart disease	3086 (18%)	2923 (17%)	2305 (17%)	0.01	0.01
Heart failure	1574 (9%)	1430 (8%)	1367 (10%)	0.04	0.03
Heart Valve Disease	356 (2%)	376 (2%)	275 (2%)	0.01	0.01
Coronary revascularization	1633 (9%)	1583 (9%)	1262 (9%)	< 0.01	< 0.01
Atrial fibrillation	1709 (10%)	1657 (10%)	1301 (10%)	< 0.01	< 0.01
Other arrhythmias	1209 (7%)	1159 (7%)	1109 (8%)	0.03	0.03
Peripheral vascular disease	640 (4%)	498 (3%)	675 (5%)	0.07	0.07
Stroke	957 (5%)	888 (5%)	799 (6%)	0.02	0.02
Other cerebrovascular disease	1031 (6%)	1001 (6%)	861 (6%)	0.01	0.02
Diabetes complications	9446 (54%)	9057 (53%)	6913 (51%)	0.04	0.04
Cancer	1862 (11%)	1847 (11%)	1534 (11%)	0.01	0.01
COPD	1141 (6%)	1071 (6%)	1215 (9%)	0.07	0.06
Renin-angiotensin inhibitors	10,883 (62%)	10,832 (63%)	8669 (64%)	0.02	0.02
Mineralocorticoid antagonists	855 (5%)	785 (5%)	685 (5%)	0.01	0.01
Thiazides	746 (4%)	686 (4%)	740 (5%)	0.04	0.05
Loop diuretics	2005 (11%)	1865 (11%)	1704 (12%)	0.03	0.02
Beta blockers	6573 (37%)	6467 (38%)	5341 (39%)	0.02	0.02
Calcium channel blockers	5128 (29%)	5012 (29%)	4121 (30%)	0.01	0.02
Metformin	13,354 (76%)	13,031 (76%)	10,357 (76%)	0.01	0.01
Insulin	3627 (21%)	3476 (20%)	2681 (20%)	0.02	0.01
Sulfonylurea	4612 (26%)	4468 (26%)	3206 (23%)	0.04	0.04
Other diabetes medications	944 (5%)	890 (5%)	345 (3%)	0.10	0.09
NSAID	2710 (15%)	2686 (16%)	2028 (15%)	0.02	0.01
Lipid-lowering medications	9747 (55%)	9708 (57%)	7563 (55%)	0.02	0.02
PPI	3914 (22%)	3788 (22%)	3158 (23%)	0.02	0.02

Baseline characteristics of DPP-4i, GLP1-RA and SGLT2i after propensity score using inverse probability treatment weighting (IPTW) with standardized mean differences (SMD). The fifth column contains the SMDs when DPP4i is the reference, the sixth column contains the SMDs when GLP1-RA is the reference. Continuous variables are presented as median (interquartile range), whereas categorical variables are presented as *n* (%)

*DPP-4i* dipeptidyl peptidase-4 inhibitor, *GLP1-RA* glucagon-like peptide-1 receptor agonist, *SGLT2i* sodium-glucose cotransporter-2 inhibitor, *Ref* reference, *COPD* chronic obstructive pulmonary disease, renin–angiotensin inhibitors, angiotensin-converting enzyme (ACE) inhibitors and angiotensin receptor blockers (ARBs), *NSAID* nonsteroidal anti-inflammatory drug, *PPI* proton pump inhibitor, *SMD* standardized mean difference

During a median follow-up of 2.5 (IQR 0.96, 4.84) years, 1411 (8.1%) participants experienced AKI (Table 2). Users of SGLT2i exhibited the lowest crude incidence rate of AKI (18.3 [CI 95% 14.1–23.4] per 1000 person-years), followed by GLP1-RA users (22.5 [CI 95% 19.9–25.3] per 1000 person-years) and DPP-4i users (26.6 [CI 95% 25–28.2] per 1000 person-years). The weighted absolute risk of AKI at 3 years was 5.79% [95% CI 3.63–8.52] for the SGLT2i group, 7.03% [95% CI 5.69–8.69] for the GLP1-RA group and 7.00% [95% CI 6.43–7.58] for the DPP-4i group. In weighted Cox regression, and compared to DPP-4i, risks of AKI were similar for GLP1-RA (0.98 [CI 95% 0.82–1.18]) or SGLT2i (0.73 [CI 95% 0.45–1.16]). Although broad confidence intervals rendered associations statistically non-significant, risk magnitudes were lowest for patients on SGLT2i. Compared to GLP1-RA, the risk of AKI for patients started on SGLT2i did not differ, with a weighted HR of 0.74 [CI 95% 0.46–1.19].

**Table 2:** Number of AKI events, incidence rates, absolute risks, absolute risk differences, and hazard ratios associated between novel glucose-lowering drugs

	Number of events	Number of person-years	Incidence rate per 1000 person-years^a^ (95% CI)	3-Year absolute risk^b^ (95% CI)	3-year ARD^b^ (95% CI)	Crude HR (95% CI)	Adjusted HR^b^ (95% CI)	Crude HR (95% CI)	Adjusted HR^b^ (95% CI)
Overall	1411	56,082	25.2 (23.9, 26.5)	6.71% (5.89, 7.63)	–	–	–	–	–
DPP-4i	1070	40,284	26.6 (25.0, 28.2)	7.00% (6.43, 7.58)	Ref	Ref	Ref	1.19 (1.04, 1.36)	1.02 (0.85, 1.21)
GLP1-RA	277	12,302	22.5 (19.9, 25.3)	7.03% (5.69, 8.69)	− 0.03% (− 1.41, 1.73)	0.84 (0.74, 0.96)	0.98 (0.82, 1.18)	Ref	Ref
SGLT2i	64	3496	18.3 (14.1, 23.4)	5.79% (3.63, 8.52)	− 1.21% (− 3.48, 1.65)	0.66 (0.51, 0.85)	0.72 (0.45, 1.16)	0.78 (0.6, 1.03)	0.74 (0.46, 1.19)

*AKI* acute kidney injury, *DPP-4i* dipeptidyl peptidase-4 inhibitor, *GLP1-RA* glucagon-like peptide-1 receptor agonist, *SGLT2i*, sodium-glucose cotransporter-2 inhibitor, *CI* confidence interval, *Ref*, reference, *ARD* absolute risk difference, *HR* hazard ratio

^a^Crude incidence rate before propensity score weighting

^b^Analysis was weighted for the following 60 variables using inverse probability treatment weighting (IPTW): age, sex, index year, eGFR, CKD stage, Hba1c, education, acute coronary syndrome, other ischemic heart disease, heart failure, heart valvular disease, coronary revascularization, other cardiac surgery, atrial fibrillation, other arrhythmias, peripheral vascular disease, stroke, other cerebrovascular disease, diabetes complications, cancer, chronic obstructive pulmonary disease, other chronic pulmonary disease, venous thromboembolism, liver disease, pancreatitis, rheumatic disease, fracture, hyperkalemia, psychiatric disorder, concomitant use of renin-angiotensin system inhibitors, mineralocorticoid receptor antagonists, thiazide, loop diuretic, beta blocker, calcium channel blockers, digoxin, nitrate, metformin, insulin, sulfonylurea, other diabetic medication, non-steroidal anti-inflammatory drugs, lipid-lowering medications, proton pump inhibitor, anti-platelet medication, anticoagulant medication, antidepressant, anxiolytic medication, beta2 agonist inhalation, anticholinergic inhalation, glucocorticoid inhalation, oral glucocorticoid, opioid, total medications, hospitalization (cardiovascular, type 2 DM-related or other cause), outpatient specialist utilization (cardiovascular, type 2 DM or other cause). In addition, calendar year and CKD stage were included as additional covariates in the weighted Cox regression

Sensitivity analyses applying overlapping weights yielded similar results to our main analysis (Supplemental Table S6), with a HR for AKI of 0.89 [95% CI 0.66–1.20] among SGLT2i users compared to DPP-4i, and 0.79 [95% CI 0.57–1.09] compared to GLP1-RA.

## Discussion

Concerns about the safety of SGLT2i have been raised by the US FDA [11] after reviewing voluntary reporting of cases in the “Adverse Event Reporting System (FAERS)”. Such possible risks were not noted by pivotal clinical trials [3, 4, 6, 7]. However, AKI is a rare outcome, and these clinical trials were underpowered to assess it robustly. Post-marketing surveillance studies in the heterogeneous routine clinical care are thus a necessary complement to assess the safety of SGLT2i. In this study, we compared the risk of detected AKI among users of novel glucose-lowering drugs in Stockholm´s routine healthcare. We observed that after balancing for an extensive range of confounders, AKI occurrence did not statistically differ between therapies. Instead, we observed numerically lower AKI risk magnitudes among users of SGLT2i compared to DPP4i and GLP1-RA. Results were robust to alternative methods of weighting for confounders and, collectively, add to growing evidence on the safety of initiating SGLT2i in adults with T2DM.

We studied and compared the health trajectories of 17,407 individuals with T2DM initiating these novel oral diabetes medications in our region and can now provide results that agree with and expand previous observations. Two administrative studies from Sweden and Denmark found that SGLT2i were associated with a lower risk of AKI compared with DPP-4 inhibitors (HR, 0.41 [95% CI, 0.32–0.52]) and GLP-1RA (HR, 0.69 [95% CI, 0.45–1.05]) [16, 18]. More recently, Zhuo et al. [17] reported a lower risk for SGLT2i vs DPP4i (HR 0.71, [95% CI 0.65–0.76]) and SGLT2i vs GLP1-RA groups (HR 0.81, [95% CI 0.75–0.87]) among Medicare users in the US. Important limitations of these studies were that they lacked information on the patient’s eGFR and that they identified AKI events through ICD diagnostic codes, which have low sensitivity. However, together with our findings, they provide reassurance on the safety of SGLT2i with respect to the risk of AKI and suggest that SGLT2i may prevent AKI events compared with alternative diabetes treatments. The mechanisms by which SGLT2i may protect against AKI compared with other glucose-lowering agents are still under investigation [26], and may include attenuation of glomerular hyperfiltration [27, 28], possibly due to tubular-glomerular feedback mechanisms through increased sodium delivery to macula densa [28]. Other postulated mechanisms involve altered renal oxygen homeostasis [29], reduced renal inflammation and decreased ischemic proximal tubular cell injury [30].

Our study has several strengths. Regarding the exposure, we ascertained it through pharmacy dispensations, which are better indicators of therapy initiation than prescription claims, in a country that provides universal healthcare access with almost all medication costs financed by the Government. This reduces healthcare access bias and increases face validity. However, we cannot guarantee that the medications dispensed at the pharmacy were taken by the patient. Regarding the outcome, we ascertained AKI events through both inpatient diagnostic codes and transient creatinine elevations according to KDIGO classification. This is important because AKI diagnoses are specific, but have low sensitivity [31]. By applying an active comparator new user design, we were able to minimize both confounding by indication and time-related bias, and we could identify and account for a large battery of confounders through propensity score weighting.

Our study also had limitations. Although our data source covers the healthcare of Stockholm region during 2008–2018, our study was limited in both size and length of follow-up and was also underpowered to compare single SGLT2i subclasses. This data represents AKI risks associated to these therapies in Stockholm between 2008 and 2018. Since then, new indications for SGLT2i have been approved, including a lower eGFR [3, 4, 32] and use in patients with CVD [33]. Future studies should confirm our observations in the broader use of this medication. Some of the currently most popular SGLT2i, such as dapagliflozin, empagliflozin and canagliflozin were approved by the European Medicines Agency (EMA) in 2012–2014, [34–36], and patients who started SGLT2i during the introduction of these therapies may differ in severity or characteristics from those who start today. Our study had a median follow-up of 2.5 years and it could be argued that, with longer duration of treatment, AKI signals may be more prominent. However, more than half of the AKI cases reported to the Adverse Event Reporting System (58 cases) occurred within 1 month of SGLT2i initiation. As in any observational study, residual and unknown confounding may still exist, and our results only report associations, not causal effects.

To conclude, in this region-representative study of routine care patients with T2DM initiating novel glucose-lowering drugs, we observed similar occurrence of AKI between therapies. Although not-statistically significant, we also observed a lower AKI risk associated to SGLT2i compared with DPP-4 inhibitor and GLP-1RA initiation. Our data thus offer support to mounting evidence on the safety of initiating SGLT2i.

## Supplementary Information

Below is the link to the electronic supplementary material.

Supplementary file1 (DOCX 747 kb)

## Data Availability

Datasets used and/or analyzed during the current study are available from the corresponding author on reasonable request for collaborative research that is compliant with GDPR regulations.
