# Supplementary material for: Novel glucose-lowering drugs and the risk of acute kidney injury in routine care; the Stockholm CREAtinine Measurements (SCREAM) project
Source: J Nephrol. 2022 Dec 2;36(3):705–11. doi: 10.1007/s40620-022-01505-8 (PMC10090010; doi:10.1007/s40620-022-01505-8)
Supplement: Supplementary file 1 — Supplementary file1 (DOCX 747 kb) [file 40620_2022_1505_MOESM1_ESM.docx]

SUPPLEMENTARY MATERIAL

Table S1. Definition of covariates.

Table S2. Definition of outcome.
Table S3. Selected baseline characteristics before and after propensity score weighting (IPTW).

Table S4. Additional baseline characteristics before and after propensity score weighting (IPTW).

Table S5. Selected baseline characteristics, before and after overlap weighting.

Table S6. Incidence rate, absolute risk, crude, and adjusted hazard ratio after overlap weighting.

Table S7. Additional baseline covariates, before and after overlap weighting

Figure S1. Selection of study population flowchart.

Figure S2. Study design diagram.

**Table S1.** Definition of covariates.

| **Treatment (exposure)** |  |
| --- | --- |
| Dipeptidyl peptidase-4 inhibitors (DPP-4i) | A10BH, A10BH01, A10BH02, A10BH03, A10BH04, A10BH05, A10BH06, A10BH07, A10BH08, A10BD07, A10BD08, A10BD09, A10BD10, A10BD11, A10BD12, A10BD13 |
| Glucagon-like peptide-1 agonists (GLP1-RA) | A10BJ01, A10BJ02, A10BJ03, A10BJ04, A10BJ05, A10BJ06, A10BJ07, A10AE56, A10AE54 |
| Sodium-glucose cotransporter-2 inhibitors (SGLT2i) | A10BK, A10BK01, A10BK02, A10BK03, A10BK04, A10BK05, A10BK06, A10BK07, A10BD15, A10BD16, A10BD20 |
| **Demographics:** |  |
| Age | Age at initiation |
| Sex | Registered sex |
| Calendar year | Index year of cohort entry |
| Education | Values according SUN2000niva_old.  1 = Compulsory school, < 9 years (did not complete compulsory education)  2 = Compulsory school, 9 years (completed compulsory education)  3 = Secondary school (upper secondary, 2 years)  4 = Secondary school (upper secondary, 3 years)  5 = University (college/university < 3 years)  6 = University (college/university >= 3 years)  7 = University (research education) |
| **Labs:** |  |
| eGFR_6 months | Average during previous 6 months before CED |
| eGFR_12 months | Average during previous 12 months before CED |
| CKD stage_6 months | G1-G5, based on average eGFR during previous 6 months before CED |
| CKD stage_12 months | G1-G5, based on average eGFR during previous 12 months before CED |
| HbA1c_ 6 months | Average HbA1c during previous 6 months before CED |
| HbA1c_12 months | Average HbA1c during previous 12 months before CED |
|  |  |
| **Medications previous 6 months:** | **ATC code** |
| RAS inhibitor (Angiotensin converting enzyme inhibitors and Angiotensin II receptor blockers) | C09A, C09B, C09C, C09D |
| Calcium channel blocker | C08 |
| Thiazide | C03A |
| MRA | C03DA01, C03DA04 |
| Loop diuretic | C03C |
| Beta blockers | C07 |
| Digoxin | C01AA05 |
| Nitrate | C01DA |
| Platelet inhibitors | B01AC |
| Anticoagulants | B01AA, B01AE07, B01AF, B01AX05 |
| Lipid lowering drugs | C10 |
| Antidepressants | N06A |
| Antipsychotic | N05A |
| Anxiolytic hypnotic sedative | N05B, N05C |
| Beta2 agonist inhalant | R03AC |
| Anticholinergic inhalant | R03BB |
| Glucocorticoid inhalant | R03BA, R03AK |
| Oral glucocorticoid | H02AB |
| NSAID | M01A |
| Opioid | N02A |
| Proton pump inhibitors | A02BC |
| Metformin | A10BA02, A10BD02, A10BD03, A10BD05, A10BD07, A10BD08, A10BD10, A10BD11, A10BD13, A10BD14, A10BD15, A10BD16, A10BD20 |
| Insulin | A10AB, A10AC, A10AD, A10AE |
| Sulfonylurea | A10BB, A10BD01, A10BD02, A10BD04, A10BD06 |
| Other diabetic drugs | A10BF01, A10BG, A10BD03, A10BD04, A10BD05, A10BD06, A10BD09, A10BD14, A10BX |
| Total number of unique medications previous 6 months | 1 = “0-5”, 2 = “6-10”, 3 = “11-15”, 4 = “>15” |
| **Comorbidities:** | **ICD-10 code** |
| Acute coronary syndrome | I200, I21, I22 |
| Other ischemic heart disease | I201, I208, I209, I24, I25 |
| Heart failure | I110, I130, I132, I50 |
| Valvular disorder | I34, I35, I36, I37 |
| Stroke | I60, I61, I62, I63, I64 |
| Other cerebrovascular disease | I65, I66, I67, I68, I69, G450, G451, G452, G453, G458, G459, G46 |
| Atrial fibrillation | I48 |
| Other arrhythmias | I44, I45, I46, I47, I49 |
| Coronary revascularization | FNG00, FNG02, FNG05, FNA, FNB, FNC, FND, FNE, FNF, FNG, FNH, FNJ, FNK, FNW |
| Other cardiac operation | F except FNG00, FNG02, FNG05, FNA, FNB, FNC, FND, FNE, FNF, FNG, FNH, FNJ, FNK, FNW |
| Peripheral vascular disease | I70, I72, I73 |
| Diabetes complication | E110-E116, E118, E130-E136, E138, E140-E148, E160, E161, E162, G990, G590, G632, H280, H358, H360, M142, M146 |
| Chronic obstructive pulmonary disease (COPD) | J44 |
| Other lung disease | I270, I272, I278, I279, J40, J41, J42, J43, J44, J45, J46, J47, J60, J61, J62, J63, J64, J65, J66, J67, J68, J69, J70, J84, J92, J96, J982, J983 |
| Venous thromboembolism | I26, I801, I802, I803, I808, I809, I81, I820, I822, I823, I828, I829 |
| Cancer | C01-C26, C30-C34, C37-C41, C43, C45-C58, C60-C76, C81-C86, C88, C90-C97 |
| Liver disease | B18, I850, I859, I982, K70, K71, K72, K73, K74, K75, K76, K77 |
| Rheumatic disease | M05, M06, M07, M08, M09, M30, M31, M32, M33, M34, M351, M353, M45 |
| Psychiatric disorder | F04, F05, F06, F07, F08, F09, F10, F2[0-9], F3[-9], F4[0-9], F5[0-9], F6[0-9], F7[0-9], F8[0-9], F9[0-9] |
| Pancreatitis | K85, K860, K861 |
| Fracture | S020, S021, S022, S023, S024, S026, S027, S028, S029, S12, S22, S32, S42, S52, S62, S72, S82, S92, T02, T08, T10, T12, M484, M485, M843 |
| Hyperkalemia | E875 |
| Previous acute kidney injury | N17 |
| **Healthcare utilization (previous year)** | **ICD-10 code** |
| **Outpatient** |  |
| Cardiovascular cause | I, G45, G46, H341 (primary position) |
| Diabetes mellitus type 2 complications | E11 (primary position) |
| Other cause | Codes other than I, G45, G46, H341  (primary position) |
| **Hospitalization** |  |
| Cardiovascular cause | I, G45, G46, H341 (primary position) |
| Diabetes mellitus type 2 complications | E11 (primary position) |
| Other cause | Codes other than I, G45, G46, H341  (primary position) |

**Table S2.** Definition of outcome.

| **Outcomes** | **Definition** | **End of follow-up** |
| --- | --- | --- |
| **Acute kidney Injury** | |  |
| AKI | - Outpatient or in-hospital with ICD-10 code N17 - Elevation of kidney function during hospitalization, defined in 3 stages:   - Stage 1: Creatinine elevation 1.5-1.9 times higher than baseline creatinine in the first 7 days after admission or creatinine increase of ≥ 26.5 µmol/L within 48 hours   - Stage 2: Creatinine elevation 2.0-2.9 times higher than baseline creatinine in the first 7 days after admission   - Stage 3: Creatinine increase of ≥ 353.6 µmol/L within 48 hours or creatinine elevation ≥3 times higher than baseline creatinine or need for acute dialysis (defined by procedure codes DR015 and DR023)   Baseline creatinine was defined as average of outpatient measurements during the period 12 months to 7 days prior hospital admission | 31^st^ December 2018 |

**Table S3.** Selected baseline characteristics of DPP-4i, GLP1-RA and SGLT2i before and after propensity score weighting.

|  | **Before weighting** | | | | | **After weighting** | | | | |
| --- | --- | --- | --- | --- | --- | --- | --- | --- | --- | --- |
|  | **DPP-4i** | **GLP1** | **SGLT2i** | **SMD DPP-4i Ref.** | SMD  GLP1 Ref. | **DPP-4i** | **GLP1** | **SGLT2i** | **SMD  DPP-4i Ref.** | SMD   GLP1 Ref. |
| **Number of individuals** | 10605 | 4448 | 2354 |  |  | 17573 | 17082 | 13645 |  |  |
| **Age, median [IQR]** | 65  [57, 73] | 59  [51, 67] | 63  [56, 71] | **0.35** | 0.35 | 63  [54, 72] | 63  [55, 71] | 64  [56, 72] | **0.08** | 0.07 |
| **Women** | 4052 (38%) | 1897  (43%) | 768  (33%) | **0.14** | 0.14 | 6784  (39%) | 6408 (38%) | 5384 (39%) | **0.03** | 0.03 |
| **eGFR, (ml/min/1.73m^2^), [IQR]** | 87  [69, 99] | 93  [78, 103] | 90  [77, 100] | **0.21** | 0.21 | 89  [72, 100] | 90  [74, 100] | 87  [69, 99] | **0.08** | 0.07 |
| **CKD stage** |  |  |  | **0.29** | 0.29 |  |  |  | **0.14** | 0.13 |
| **G1 (≥ 90 ml/min/1.73 m^2^)** | 4936 (47%) | 2591  (58%) | 1248  (53%) |  |  | 8885  (51%) | 8787 (51%) | 6277 (46%) |  |  |
| **G2 (60-89 ml/min/1.73 m^2^)** | 3941 (37%) | 1468  (33%) | 970  (41%) |  |  | 6396  (36%) | 6162 (36%) | 5104 (37%) |  |  |
| **G3a (45-59 ml/min/1.73 m^2^)** | 1035 (10%) | 256  (6%) | 110  (5%) |  |  | 1416  (8%) | 1201 (7%) | 1293 (9%) |  |  |
| **G3b (30-44 ml/min/1.73 m^2^)** | 587 (6%) | 119  (3%) | 26  (1%) |  |  | 754  (4%) | 803  (5%) | 971 (7%) |  |  |
| **G4 (15-29 ml/min/1.73 m^2^)** | 106 (1%) | 14  (0%) | 0  (0%) |  |  | 123  (1%) | 128  (1%) | 0  (0%) |  |  |
| **Hba1c (mmol/mol)** | 60  [53, 70] | 64  [54, 77] | 62  [54, 73] | **0.12** | 0.12 | 61  [54, 72] | 62  [54, 74] | 61  [52, 72] | **0.04** | 0.04 |
| **Education** |  |  |  | **0.09** | 0.09 |  |  |  | **0.07** | 0.07 |
| **Compulsory school** | 2779 (26%) | 965 (22%) | 559 (24%) |  |  | 4400  (25%) | 4290 (25%) | 3676 (27%) |  |  |
| **Secondary school** | 4538 (43%) | 2044 (46%) | 1036 (44%) |  |  | 7647  (44%) | 7413 (43%) | 5553 (41%) |  |  |
| **University** | 3028 (29%) | 1375 (31%) | 719 (31%) |  |  | 5160  (29%) | 4910 (29%) | 4188 (31%) |  |  |
| **Missing** | 260 (2%) | 64  (1%) | 40  (2%) |  |  | 366  (2%) | 468  (3%) | 228  (2%) |  |  |
| **Acute coronary syndrome** | 1022 (10%) | 354  (8%) | 387 (16%) | **0.17** | 0.17 | 1737 (10%) | 1722 (10%) | 1335 (10%) | **0.01** | 0.01 |
| **Other ischemic heart disease** | 1878 (18%) | 631 (14%) | 566 (24%) | **0.17** | 0.17 | 3086 (18%) | 2923 (17%) | 2305 (17%) | **0.01** | 0.01 |
| **Heart failure** | 954 (9%) | 353  (8%) | 227 (10%) | **0.04** | 0.04 | 1574 (9%) | 1430 (8%) | 1367 (10%) | **0.04** | 0.03 |
| **Heart valvular disease** | 249 (2%) | 62  (1%) | 46  (2%) | **0.05** | 0.05 | 356  (2%) | 376  (2%) | 275 (2%) | **0.01** | 0.01 |
| **Coronary revascularization** | 958 (9%) | 342  (8%) | 366 (16%) | **0.17** | 0.17 | 1633 (9%) | 1583 (9%) | 1262 (9%) | **<0.01** | <0.01 |
| **Atrial fibrillation** | 1100 (10%) | 344  (8%) | 247 (10%) | **0.06** | 0.06 | 1709 (10%) | 1657 (10%) | 1301 (10%) | **<0.01** | <0.01 |
| **Other arrhythmias** | 751 (7%) | 261  (6%) | 192  (8%) | **0.06** | 0.06 | 1209 (7%) | 1159 (7%) | 1109 (8%) | **0.03** | 0.03 |
| **Peripheral vascular disease** | 420 (4%) | 133  (3%) | 96  (4%) | **0.04** | 0.04 | 640  (4%) | 498  (3%) | 675 (5%) | **0.07** | 0.07 |
| **Stroke** | 635 (6%) | 202  (5%) | 144  (6%) | **0.05** | 0.05 | 957  (5%) | 888  (5%) | 799 (6%) | **0.02** | 0.02 |
| **Other cerebrovascular disease** | 670 (6%) | 241  (5%) | 146  (6%) | **0.03** | 0.03 | 1031 (6%) | 1001 (6%) | 861 (6%) | **0.01** | 0.02 |
| **Diabetes complications** | 5771 (54%) | 2321 (52%) | 1205 (51%) | **0.04** | 0.04 | 9446 (54%) | 9057 (53%) | 6913 (51%) | **0.04** | 0.04 |
| **Cancer** | 1255 (12%) | 365  (8%) | 241 (10%) | **0.08** | 0.08 | 1862 (11%) | 1847 (11%) | 1534 (11%) | **0.01** | 0.01 |
| **COPD** | 668 (6%) | 308  (7%) | 154  (7%) | **0.02** | 0.02 | 1141 (6%) | 1071 (6%) | 1215 (9%) | **0.07** | 0.06 |
| **Renin-angiotensin inhibitors** | 6496 (61%) | 2829 (64%) | 1535 (65%) | **0.05** | 0.05 | 10883 (62%) | 10832 (63%) | 8669 (64%) | **0.02** | 0.02 |
| **Mineral- corticoid antagonists** | 479 (5%) | 237  (5%) | 117  (5%) | **0.03** | 0.03 | 855  (5%) | 785  (5%) | 685 (5%) | **0.01** | 0.01 |
| **Thiazides** | 460 (4%) | 212  (5%) | 79  (3%) | **0.05** | 0.05 | 746  (4%) | 686  (4%) | 740 (5%) | **0.04** | 0.05 |
| **Loop diuretics** | 1240 (12%) | 524 (12%) | 178  (8%) | **0.10** | 0.10 | 2005 (11%) | 1865 (11%) | 1704 (12%) | **0.03** | 0.02 |
| **Beta blockers** | 4061 (38%) | 1562 (35%) | 918 (39%) | **0.05** | 0.05 | 6573 (37%) | 6467 (38%) | 5341 (39%) | **0.02** | 0.02 |
| **Calcium channel blockers** | 3086 (29%) | 1306 (29%) | 686 (29%) | **<0.01** | <0.01 | 5128 (29%) | 5012 (29%) | 4121 (30%) | **0.01** | 0.02 |
| **Metformin** | 8088 (76%) | 3284 (74%) | 1899 (81%) | **0.11** | 0.11 | 13354 (76%) | 13031 (76%) | 10357 (76%) | **0.01** | 0.01 |
| **Insulin** | 1269 (12%) | 1678 (38%) | 470 (20%) | **0.41** | 0.41 | 3627  (21%) | 3476 (20%) | 2681 (20%) | **0.02** | 0.01 |
| **Sulfonylurea** | 2952 (28%) | 992 (22%) | 583 (25%) | **0.09** | 0.09 | 4612 (26%) | 4468 (26%) | 3206 (23%) | **0.04** | 0.04 |
| **Other diabetes medications** | 660 (6%) | 191  (4%) | 55  (2%) | **0.13** | 0.13 | 944  (5%) | 890  (5%) | 345 (3%) | **0.10** | 0.09 |
| **NSAID** | 1598 (15%) | 747 (17%) | 303 (13%) | **0.07** | 0.07 | 2710 (15%) | 2686 (16%) | 2028 (15%) | **0.02** | 0.01 |
| **Lipid-lowering medications** | 5966 (56%) | 2361 (53%) | 1373 (58%) | **0.07** | 0.07 | 9747 (55%) | 9708 (57%) | 7563 (55%) | **0.02** | 0.02 |
| **PPI** | 2315 (22%) | 991 (22%) | 523 (22%) | **0.01** | 0.01 | 3914 (22%) | 3788 (22%) | 3158 (23%) | **0.02** | 0.02 |

Selected baseline characteristics before and after propensity score using inverse probability treatment weighting (IPTW) incl. standardized mean difference (SMD). The fifth and tenth columns contain the SMDs when DPP-4i is the reference, the sixth and eleventh columns contain the SMDs when GLP1-RA is the reference. Continuous variables are presented as median (interquartile range), whereas categorical variables are presented as n (%).
DPP-4i, dipeptidyl peptidase-4 inhibitor; GLP1-RA/GLP1, glucagon-like peptide-1 receptor agonist; SGLT2i, sodium-glucose cotransporter-2 inhibitor; COPD, chronic obstructive pulmonary disease; Renin-angiotensin inhibitors, angiotensin-converting enzyme (ACE) inhibitors and angiotensin receptor blockers (ARBs); NSAID, nonsteroidal anti-inflammatory drug; PPI, proton pump inhibitor.

**Table S4.** Additional baseline characteristics of DPP-4i, GLP1-RA and SGLT2i before and after propensity score weighting.

|  | **Before weighting** | | | | | **After weighting** | | | | |
| --- | --- | --- | --- | --- | --- | --- | --- | --- | --- | --- |
|  | **DPP-4i** | **GLP1** | **SGLT2i** | **SMD DPP4i Ref.** | SMD GLP1 Ref. | **DPP-4i** | **GLP1** | **SGLT2i** | **SMD DPP4i Ref.** | SMD GLP1 Ref. |
| **Calendar year** |  |  |  | **0.82** | 0.82 |  |  |  | **0.55** | 0.56 |
| **2008** | 519 (5%) | 83  (2%) | 0  (0%) |  |  | 613 (3%) | 677 (4%) | 0  (0%) |  |  |
| **2009** | 517 (5%) | 53  (1%) | 0  (0%) |  |  | 568 (3%) | 362 (2%) | 0  (0%) |  |  |
| **2010** | 716 (7%) | 165 (4%) | 0  (0%) |  |  | 897 (5%) | 928 (5%) | 0  (0%) |  |  |
| **2011** | 735 (7%) | 235 (5%) | 0  (0%) |  |  | 995 (6%) | 1073 (6%) | 0  (0%) |  |  |
| **2012** | 790 (7%) | 234 (5%) | 0  (0%) |  |  | 1019 (6%) | 1054 (6%) | 0  (0%) |  |  |
| **2013** | 846 (8%) | 206 (5%) | 39  (2%) |  |  | 1106 (6%) | 1052 (6%) | 1020 (7%) |  |  |
| **2014** | 852 (8%) | 285 (6%) | 115 (5%) |  |  | 1251 (7%) | 1194 (7%) | 1437 (11%) |  |  |
| **2015** | 1186 (11%) | 519 (12%) | 205 (9%) |  |  | 1889 (11%) | 1812 (11%) | 2055 (15%) |  |  |
| **2016** | 1394 (13%) | 593 (13%) | 362 (15%) |  |  | 2306 (13%) | 2176 (13%) | 2483 (18%) |  |  |
| **2017** | 1500 (14%) | 870 (20%) | 537 (23%) |  |  | 3006 (17%) | 2991 (18%) | 2954 (22%) |  |  |
| **2018** | 1550 (15%) | 1205 (27%) | 1096 (47%) |  |  | 3921 (22%) | 3764 (22%) | 3697 (27%) |  |  |
| **Other cardiac operation** | 591 (6%) | 179 (4%) | 147 (6%) | **0.07** | 0.07 | 896 (5%) | 901 (5%) | 808 (6%) | **0.02** | 0.02 |
| **Other lung disease** | 1856 (18%) | 961 (22%) | 426 (18%) | **0.07** | 0.07 | 3313 (19%) | 3145 (18%) | 2922 (21%) | **0.05** | 0.05 |
| **Venous thrombo-embolism** | 533 (5%) | 227 (5%) | 118 (5%) | **<0.01** | <0.01 | 897 (5%) | 825 (5%) | 775 (6%) | **0.03** | 0.03 |
| **Liver disease** | 436 (4%) | 247 (6%) | 123 (5%) | **0.04** | 0.04 | 837 (5%) | 801 (5%) | 727 (5%) | **0.02** | 0.02 |
| **Pancreatitis** | 175 (2%) | 73 (2%) | 54  (2%) | **0.03** | 0.03 | 304 (2%) | 301 (2%) | 280 (2%) | **0.02** | 0.01 |
| **Rheumatic disease** | 472 (4%) | 193 (4%) | 105 (4%) | **<0.01** | <0.01 | 769 (4%) | 761 (4%) | 549 (4%) | **0.01** | 0.01 |
| **Fracture** | 1893 (18%) | 773 (17%) | 461 (20%) | **0.04** | 0.04 | 3202 (18%) | 3021 (18%) | 2695 (20%) | **0.04** | 0.03 |
| **Hyper- kalemia** | 37 (0%) | 17 (0%) | 6  (0%) | **0.02** | 0.02 | 61  (0%) | 79  (0%) | 37  (0%) | **0.02** | 0.02 |
| **Psychiatric disorder** | 3406 (32%) | 1816 (41%) | 903 (38%) | **0.12** | 0.12 | 6238 (35%) | 6048 (35%) | 5531 (41%) | **0.07** | 0.07 |
| **Digoxin** | 263 (2%) | 60 (1%) | 48  (2%) | **0.06** | 0.06 | 378 (2%) | 291 (2%) | 262 (2%) | **0.02** | 0.02 |
| **Nitrate** | 742 (7%) | 235 (5%) | 208 (9%) | **0.09** | 0.09 | 1184 (7%) | 1172 (7%) | 722 (5%) | **0.04** | 0.04 |
| **Platelet inhibitor** | 3226 (30%) | 1097 (25%) | 709 (30%) | **0.09** | 0.09 | 5047 (29%) | 4901 (29%) | 3692 (27%) | **0.02** | 0.02 |
| **Anti- coagulants** | 823 (8%) | 295 (7%) | 197 (8%) | **0.04** | 0.04 | 1312 (7%) | 1246 (7%) | 1006 (7%) | **<0.01** | 0.01 |
| **Anti- depressant** | 1268 (12%) | 771 (17%) | 300 (13%) | **0.10** | 0.10 | 2397 (14%) | 2253 (13%) | 2090 (15%) | **0.04** | 0.05 |
| **Anxiolytic** | 2034 (19%) | 881 (20%) | 389 (17%) | **0.06** | 0.06 | 3347 (19%) | 3278 (19%) | 2812 (21%) | **0.03** | 0.03 |
| **Beta2- agonist inhalant** | 626 (6%) | 374 (8%) | 167 (7%) | **0.06** | 0.06 | 1200 (7%) | 1125 (7%) | 968 (7%) | **0.01** | 0.02 |
| **Anti-cholinergic inhalant** | 306 (3%) | 133 (3%) | 71  (3%) | **0.01** | 0.01 | 503 (3%) | 471 (3%) | 541 (4%) | **0.04** | 0.04 |
| **Gluco-corticoid inhalant** | 861 (8%) | 472 (11%) | 201 (9%) | **0.06** | 0.06 | 1574 (9%) | 1530 (9%) | 1268 (9%) | **0.01** | 0.01 |
| **Oral gluco-corticoid** | 623 (6%) | 289 (6%) | 147 (6%) | **0.02** | 0.02 | 1073 (6%) | 1183 (7%) | 798 (6%) | **0.03** | 0.03 |
| **Opioid** | 1241 (12%) | 659 (15%) | 278 (12%) | **0.06** | 0.06 | 2234 (13%) | 2155 (13%) | 1666 (12%) | **0.01** | 0.01 |
| **Hospital admissions, previous year** |  |  |  |  |  |  |  |  |  |  |
| **Cardio- vascular cause** | 1341 (13%) | 474 (11%) | 333 (14%) | **0.07** | 0.07 | 2133 (12%) | 2094 (12%) | 1443 (11%) | **0.04** | 0.03 |
| **Type 2 diabetes related cause** | 1456 (14%) | 548 (12%) | 343 (15%) | **0.04** | 0.04 | 2372 (13%) | 2335 (14%) | 1820 (13%) | **0.01** | 0.01 |
| **Other cause** | 1947 (18%) | 751 (17%) | 439 (19%) | **0.03** | 0.03 | 3193 (18%) | 3155 (18%) | 2352 (17%) | **0.02** | 0.03 |
| **Outpatient utilization, previous year** |  |  |  |  |  |  |  |  |  |  |
| **Cardio-vascular cause** | 1672 (16%) | 894 (20%) | 423 (18%) | **0.08** | 0.08 | 3008 (17%) | 2769 (16%) | 1984 (15%) | **0.05** | 0.04 |
| **Type 2 diabetes cause** | 2571 (24%) | 1640 (37%) | 684 (29%) | **0.18** | 0.18 | 5101 (29%) | 4871 (29%) | 3548 (26%) | **0.05** | 0.04 |
| **Other cause** | 7148 (67%) | 3357 (75%) | 1768 (75%) | **0.12** | 0.12 | 12435 (71%) | 11944 (70%) | 10166 (75%) | **0.07** | 0.06 |
| **Total medications** |  |  |  | **0.08** | 0.08 |  |  |  | **0.06** | 0.06 |
| **1 (0-5)** | 4645 (44%) | 1814 (41%) | 1063 (45%) |  |  | 7679 (44%) | 7086 (42%) | 5795 (42%) |  |  |
| **2 (6-10)** | 4815 (45%) | 2042 (46%) | 1013 (43%) |  |  | 7852 (45%) | 8010 (47%) | 6423 (46%) |  |  |
| **3 (11-15)** | 994 (9%) | 523 (12%) | 246 (10%) |  |  | 1729 (10%) | 1702 (10%) | 1468 (11%) |  |  |
| **4 (>15)** | 151 (1%) | 69 (2%) | 32  (1%) |  |  | 332 (2%) | 192 (1%) | 170 (1%) |  |  |

Additional baseline characteristics before and after propensity score using inverse probability treatment weighting (IPTW) incl. standardized mean difference (SMD). The fifth and tenth columns contain the SMDs when DPP-4i is the reference, the sixth and eleventh columns contain the SMDs when GLP1-RA is the reference. Continuous variables are presented as median (interquartile range), whereas categorical variables are presented as n (%).
DPP-4i, dipeptidyl peptidase-4 inhibitor; GLP1-RA/GLP1, glucagon-like peptide-1 receptor agonist; SGLT2i, sodium-glucose cotransporter-2 inhibitor; COPD, chronic obstructive pulmonary disease; Renin-angiotensin inhibitors, angiotensin-converting enzyme (ACE) inhibitors and angiotensin receptor blockers (ARBs); NSAID, nonsteroidal anti-inflammatory drug; PPI, proton pump inhibitor; DM, diabetes mellitus.

**Table S5.** Baseline characteristics of DPP-4i, GLP1-RA and SGLT2i before and after overlap weighting.

|  | **DPP-4i** | **GLP1-RA** | **SGLT2i** | **SMD**  **DPP-4i**  **Ref.** | **DPP-4i** | **GLP1-RA** | **SGLT2i** | **SMD DPP-4i**  **Ref.** | **SMD**  **GLP1-RA**  **Ref.** |
| --- | --- | --- | --- | --- | --- | --- | --- | --- | --- |
| **Number of individuals** | 10605 | 4448 | 2354 |  | 264 | 266 | 261 |  |  |
| **Age (years)** | 65  [57, 73] | 59  [51, 67] | 63  [56, 71] | **0.35** | 61  [53, 70] | 61  [53, 69] | 62  [54, 69] | **0.03** | 0.02 |
| **Women** | 4052 (38%) | 1897 (43%) | 768 (33%) | **0.14** | 96  (36%) | 94  (36%) | 94  (36%) | **0.01** | 0.01 |
| **eGFR (ml/min/1.73 m^2^)** | 87  [69, 99] | 93  [78, 103] | 90  [77, 100] | **0.21** | 92  [77, 102] | 92  [79, 101] | 91  [78, 101] | **0.02** | 0.02 |
| **CKD stage** |  |  |  | **0.29** |  |  |  | **0.04** | 0.05 |
| **G1** | 4936 (47%) | 2591 (58%) | 1248 (53%) |  | 148 (56%) | 151 (57%) | 144 (55%) |  |  |
| **G2** | 3941 (37%) | 1468 (33%) | 970 (41%) |  | 100 (38%) | 99  (37%) | 101 (39%) |  |  |
| **G3a** | 1035 (10%) | 256  (6%) | 110  (5%) |  | 13  (5%) | 13  (5%) | 13  (5%) |  |  |
| **G3b** | 587 (6%) | 119  (3%) | 26  (1%) |  | 3  (1%) | 3  (1%) | 2  (1%) |  |  |
| **G4** | 106 (1%) | 14  (0%) | 0  (0%) |  | 0  (0%) | 0  (0%) | 0  (0%) |  |  |
| **Hba1c** | 60  [53, 70] | 64  [54, 77] | 62  [54, 73] | **0.12** | 62  [54, 73] | 63  [54, 75] | 62  [54, 73] | **0.01** | 0.01 |
| **Education** |  |  |  | **0.09** |  |  |  | **0.02** | 0.02 |
| **Compulsory school** | 2779 (26%) | 965 (22%) | 559 (24%) |  | 63  (24%) | 61  (23%) | 58  (22%) |  |  |
| **Secondary school** | 4538 (43%) | 2044 (46%) | 1036 (44%) |  | 116 (44%) | 119 (45%) | 117 (45%) |  |  |
| **University** | 3028 (29%) | 1375 (31%) | 719 (31%) |  | 81  (31%) | 82  (31%) | 82  (31%) |  |  |
| **Missing** | 260 (2%) | 64  (1%) | 40  (2%) |  | 4  (2%) | 4  (2%) | 4  (2%) |  |  |
| **Acute coronary syndrome** | 1022 (10%) | 354  (8%) | 387 (16%) | **0.17** | 29  (11%) | 29  (11%) | 28  (11%) | **0.01** | <0.01 |
| **Other ischemic heart disease** | 1878 (18%) | 631 (14%) | 566 (24%) | **0.17** | 48  (18%) | 49  (18%) | 46  (18%) | **0.01** | 0.01 |
| **Heart failure** | 954 (9%) | 353  (8%) | 227 (10%) | **0.04** | 22  (8%) | 21  (8%) | 21  (8%) | **0.01** | 0.01 |
| **Heart valvular disease** | 249 (2%) | 62  (1%) | 46  (2%) | **0.05** | 4  (2%) | 4  (2%) | 4  (2%) | **0.01** | 0.01 |
| **Revascular-ization** | 958 (9%) | 342  (8%) | 366 (16%) | **0.17** | 28  (10%) | 27  (10%) | 27  (10%) | **<0.01** | 0.01 |
| **Atrial fibrillation** | 1100 (10%) | 344  (8%) | 247 (10%) | **0.06** | 24  (9%) | 24  (9%) | 24  (9%) | **<0.01** | <0.01 |
| **Other arrhythmia** | 751 (7%) | 261  (6%) | 192  (8%) | **0.06** | 19  (7%) | 19  (7%) | 18  (7%) | **<0.01** | 0.01 |
| **Peripheral vascular disease** | 420 (4%) | 133  (3%) | 96  (4%) | **0.04** | 9  (3%) | 9  (3%) | 9  (3%) | **<0.01** | <0.01 |
| **Stroke** | 635 (6%) | 202  (5%) | 144  (6%) | **0.05** | 13  (5%) | 14  (5%) | 14  (5%) | **0.01** | 0.01 |
| **Other cerebrovasc-ular disease** | 670 (6%) | 241  (5%) | 146  (6%) | **0.03** | 14  (5%) | 15  (6%) | 14  (6%) | **0.01** | 0.01 |
| **Diabetes complications** | 5771 (54%) | 2321 (52%) | 1205 (51%) | **0.04** | 133 (50%) | 128 (48%) | 129 (49%) | **0.03** | 0.02 |
| **Cancer** | 1255 (12%) | 365  (8%) | 241 (10%) | **0.08** | 24  (9%) | 24  (9%) | 25  (9%) | **0.01** | <0.01 |
| **COPD** | 668 (6%) | 308  (7%) | 154  (7%) | **0.02** | 17  (6%) | 17  (7%) | 17  (7%) | **<0.01** | <0.01 |
| **Renin-angiotensin inhibitors** | 6496 (61%) | 2829 (64%) | 1535 (65%) | **0.05** | 166 (63%) | 172 (65%) | 167 (64%) | **0.02** | 0.02 |
| **Mineral- corticoid antagonists** | 479 (5%) | 237  (5%) | 117  (5%) | **0.03** | 13  (5%) | 12  (5%) | 12  (5%) | **0.01** | 0.01 |
| **Thiazides** | 460 (4%) | 212  (5%) | 79  (3%) | **0.05** | 10  (4%) | 10  (4%) | 10  (4%) | **<0.01** | <0.01 |
| **Loop diuretics** | 1240 (12%) | 524 (12%) | 178  (8%) | **0.10** | 23  (9%) | 22  (8%) | 21  (8%) | **0.01** | 0.01 |
| **Beta blockers** | 4061 (38%) | 1562 (35%) | 918 (39%) | **0.05** | 94  (36%) | 96  (36%) | 92  (35%) | **0.01** | 0.01 |
| **Calcium channel blockers** | 3086 (29%) | 1306 (29%) | 686 (29%) | **<0.01** | 78  (30%) | 78  (29%) | 78  (30%) | **0.01** | 0.01 |
| **Metformin** | 8088 (76%) | 3284 (74%) | 1899 (81%) | **0.11** | 209 (79%) | 207 (78%) | 205 (79%) | **0.01** | 0.02 |
| **Insulin** | 1269 (12%) | 1678 (38%) | 470 (20%) | **0.41** | 62  (23%) | 62  (23%) | 58  (22%) | **0.02** | 0.01 |
| **Sulfonylurea** | 2952 (28%) | 992 (22%) | 583 (25%) | **0.09** | 65  (24%) | 62  (23%) | 62  (24%) | **0.02** | 0.01 |
| **Other diabetes medications** | 660 (6%) | 191  (4%) | 55  (2%) | **0.13** | 8  (3%) | 9  (3%) | 7  (3%) | **0.02** | 0.02 |
| **NSAID** | 1598 (15%) | 747 (17%) | 303 (13%) | **0.07** | 39  (15%) | 38  (14%) | 37  (14%) | **0.01** | 0.01 |
| **Lipid-lowering medications** | 5966 (56%) | 2361 (53%) | 1373 (58%) | **0.07** | 146 (55%) | 146 (55%) | 144 (55%) | **0.01** | 0.01 |
| **PPI** | 2315 (22%) | 991 (22%) | 523 (22%) | **0.01** | 59  (22%) | 58  (22%) | 56  (22%) | **0.01** | 0.01 |

Baseline characteristics before and after overlap weighting incl. standardized mean difference (SMD). The fifth and ninth columns contain the SMDs when DPP-4i is the reference, the tenth column contains the SMDs when GLP1-RA is the reference. Continuous variables are presented as median (interquartile range), whereas categorical variables are presented as n (%).
DPP-4i, dipeptidyl peptidase-4 inhibitor; GLP-1, glucagon-like peptide-1 receptor agonist; SGLT2i, sodium-glucose cotransporter-2 inhibitor; COPD, chronic obstructive pulmonary disease; Renin-angiotensin inhibitors, angiotensin-converting enzyme (ACE) inhibitors and angiotensin receptor blockers (ARBs); NSAID, nonsteroidal anti-inflammatory drug; PPI, proton pump inhibitor.

**Table S6.** Number of AKI events, incidence rates, absolute risks, absolute risk differences, and hazard ratios associated between novel glucose-lowering drugs (overlap weighting).

|  | **Number of events** | **Number of person- years** | **Incidence rate per 1000 person- years^a^** | **3 years absolute risk^b^**  **(95% CI)** | **3 years ARD^b^**  **(95% CI)** | **Crude HR**  **(95% CI)** | **Adj. HR^b^**  **(95% CI)** | **Crude HR**  **(95% CI)** | **Adj. HR^b^**  **(95% CI)** |
| --- | --- | --- | --- | --- | --- | --- | --- | --- | --- |
| **Overall** | 1411 | 56082 | 25.2  (23.9, 26.5) | 5.36%  (4.66, 6.08) | - | - | - | - | - |
| **DPP-4i** | 1070 | 40284 | 26.6  (25, 28.2) | 5.24%  (4.37, 6.02) | **Ref.** | **Ref.** | **Ref.** | 1.19  (1.04, 1.36) | 0.88  (0.69, 1.11) |
| **GLP1-RA** | 277 | 12302 | 22.5  (19.9, 25.3) | 6.27% (5.00, 7.56) | 1.03%  (-0.45, 2.56) | 0.84  (0.74, 0.96) | 1.13  (0.90, 1.42) | **Ref.** | **Ref.** |
| **SGLT2i** | 64 | 3496 | 18.3  (14.1, 23.4) | 4.58%  (3.24, 5.96) | -0.66%  (-2.17, 1.03) | 0.66  (0.51, 0.85) | 0.89  (0.66, 1.20) | 0.78  (0.6, 1.03) | 0.79  (0.57, 1.09) |

AKI, acute kidney injury; DPP-4i, dipeptidyl peptidase-4 inhibitor; GLP-1, glucagon-like peptide-1 receptor agonist; SGLT2i, sodium-glucose cotransporter-2 inhibitor; CI, confidence interval; Ref, reference; ARD, absolute risk difference; HR, hazard ratio.
^a^ Crude incidence rate before propensity score weighting.
^b^ Analysis were adjusted for the following 60 variables using overlap weighting: age, sex, index year, eGFR, CKD stage, Hba1c, education, acute coronary syndrome, other ischemic heart disease, heart failure, heart valvular disease, coronary revascularization, other cardiac surgery, atrial fibrillation, other arrhythmias, peripheral vascular disease, stroke, other cerebrovascular disease, diabetes complications, cancer, chronic obstructive pulmonary disease, other chronic pulmonary disease, venous thromboembolism, liver disease, pancreatitis, rheumatic disease, fracture, hyperkalemia, psychiatric disorder, concomitant use of renin-angiotensin system inhibitors, mineralocorticoid receptor antagonists, thiazide, loop diuretic, beta blocker, calcium channel blockers, digoxin, nitrate, metformin, insulin, sulfonylurea, other diabetic medication, non-steroidal anti-inflammatory drugs, lipid-lowering medications, proton pump inhibitor, anti-platelet medication, anticoagulant medication, antidepressant, anxiolytic medication, beta2agonis inhalation, anticholinergic inhalation, glucocorticoid inhalation, oral glucocorticoid, opioid, total medications, hospitalization (cardiovascular, type 2 DM related or other cause), outpatient specialist utilization (cardiovascular, type 2 DM or other cause).inhalation, glucocorticoid inhalation, oral glucocorticoid, opioid, total medications, hospitalization (cardiovascular, type 2 DM related or other cause), outpatient specialist utilization (cardiovascular, type 2 DM or other cause).

# Table S7. Additional baseline characteristics of DPP-4i, GLP1-RA and SGLT2i before and after overlap weighting.

|  | **Before weighting** | | | | | **After weighting** | | | | |
| --- | --- | --- | --- | --- | --- | --- | --- | --- | --- | --- |
|  | **DPP-4i** | **GLP1** | **SGLT2i** | **SMD  DPP-4i Ref.** | SMD GLP1 Ref. | **DPP-4i** | **GLP1** | **SGLT2i** | **SMD DPP-4i Ref.** | SMD GLP1 Ref. |
| **Calendar year** |  |  |  | **0.82** | 0.82 |  |  |  | **0.10** | 0.06 |
| **2008** | 519 (5%) | 83  (2%) | 0  (0%) |  |  | 0  (0%) | 0  (0%) | 0  (0%) |  |  |
| **2009** | 517 (5%) | 53  (1%) | 0  (0%) |  |  | 0  (0%) | 0  (0%) | 0  (0%) |  |  |
| **2010** | 716 (7%) | 165  (4%) | 0  (0%) |  |  | 0  (0%) | 0  (0%) | 0  (0%) |  |  |
| **2011** | 735 (7%) | 235  (5%) | 0  (0%) |  |  | 1  (0%) | 1  (0%) | 0  (0%) |  |  |
| **2012** | 790 (7%) | 234  (5%) | 0  (0%) |  |  | 1  (0%) | 1  (0%) | 0  (0%) |  |  |
| **2013** | 846 (8%) | 206  (5%) | 39  (2%) |  |  | 6  (2%) | 6  (2%) | 5  (2%) |  |  |
| **2014** | 852 (8%) | 285 (6%) | 115  (5%) |  |  | 15  (6%) | 15  (6%) | 15  (6%) |  |  |
| **2015** | 1186 (11%) | 519 (12%) | 205  (9%) |  |  | 27  (10%) | 28  (11%) | 29  (11%) |  |  |
| **2016** | 1394 (13%) | 593 (13%) | 362 (15%) |  |  | 42  (16%) | 43  (16%) | 44  (17%) |  |  |
| **2017** | 1500 (14%) | 870 (20%) | 537 (23%) |  |  | 66  (25%) | 66  (25%) | 65  (25%) |  |  |
| **2018** | 1550 (15%) | 1205 (27%) | 1096 (47%) |  |  | 104 (40%) | 105 (39%) | 104 (40%) |  |  |
| **Other cardiac operation** | 591 (6%) | 179 (4%) | 147  (6%) | **0.07** | 0.07 | 12  (5%) | 13  (5%) | 13  (5%) | **0.01** | 0.01 |
| **Other lung disease** | 1856 (18%) | 961 (22%) | 426 (18%) | **0.07** | 0.07 | 50  (19%) | 52  (20%) | 50  (19%) | **0.01** | 0.01 |
| **Venous thromboembolism** | 533 (5%) | 227 (5%) | 118  (5%) | **<0.01** | <0.01 | 14  (5%) | 14  (5%) | 14  (5%) | **<0.01** | <0.01 |
| **Liver disease** | 436 (4%) | 247 (6%) | 123  (5%) | **0.04** | 0.04 | 15  (6%) | 14  (5%) | 15  (6%) | **0.01** | 0.01 |
| **Pancreatitis** | 175 (2%) | 73 (2%) | 54  (2%) | **0.03** | 0.03 | 5  (2%) | 5  (2%) | 6  (2%) | **<0.01** | <0.01 |
| **Rheumatic disease** | 472 (4%) | 193 (4%) | 105  (4%) | **<0.01** | <0.01 | 12  (4%) | 12  (4%) | 11  (4%) | **0.01** | <0.01 |
| **Fracture** | 1893 (18%) | 773 (17%) | 461 (20%) | **0.04** | 0.04 | 51  (19%) | 51  (19%) | 50  (19%) | **0.01** | 0.01 |
| **Hyper-kalemia** | 37 (0%) | 17 (0%) | 6  (0%) | **0.02** | 0.02 | 1  (0%) | 1  (0%) | 1  (0%) | **0.01** | 0.01 |
| **Psychiatric disorder** | 3406 (32%) | 1816 (41%) | 903 (38%) | **0.12** | 0.12 | 105 (40%) | 107 (40%) | 104 (40%) | **0.01** | 0.01 |
| **Digoxin** | 263 (2%) | 60 (1%) | 48  (2%) | **0.06** | 0.06 | 4  (2%) | 5  (2%) | 4  (2%) | **0.01** | 0.01 |
| **Nitrate** | 742 (7%) | 235 (5%) | 208  (9%) | **0.09** | 0.09 | 18  (7%) | 16  (6%) | 16  (6%) | **0.02** | 0.01 |
| **Platelet inhibitor** | 3226 (30%) | 1097 (25%) | 709 (30%) | **0.09** | 0.09 | 68  (26%) | 68  (26%) | 65  (25%) | **0.01** | 0.01 |
| **Anti- coagulants** | 823 (8%) | 295 (7%) | 197  (8%) | **0.04** | 0.04 | 19  (7%) | 21  (8%) | 20  (8%) | **0.02** | 0.02 |
| **Anti-depressant** | 1268 (12%) | 771 (17%) | 300 (13%) | **0.10** | 0.10 | 37  (14%) | 38  (14%) | 38  (14%) | **0.01** | 0.01 |
| **Anxiolytic** | 2034 (19%) | 881 (20%) | 389 (17%) | **0.06** | 0.06 | 46  (17%) | 48  (18%) | 45  (17%) | **0.02** | 0.01 |
| **Beta2- agonist inhalant** | 626 (6%) | 374 (8%) | 167  (7%) | **0.06** | 0.06 | 19  (7%) | 20  (8%) | 19  (7%) | **0.01** | 0.01 |
| **Anti-cholinergic inhalant** | 306 (3%) | 133 (3%) | 71  (3%) | **0.01** | 0.01 | 7  (3%) | 8  (3%) | 8  (3%) | **0.02** | 0.02 |
| **Gluco-corticoid inhalant** | 861 (8%) | 472 (11%) | 201  (9%) | **0.06** | 0.06 | 23  (9%) | 25  (9%) | 23  (9%) | **0.02** | 0.02 |
| **Oral gluco-corticoid** | 623 (6%) | 289 (6%) | 147  (6%) | **0.02** | 0.02 | 16  (6%) | 17  (6%) | 16  (6%) | **0.01** | 0.02 |
| **Opioid** | 1241 (12%) | 659 (15%) | 278 (12%) | **0.06** | 0.06 | 34  (13%) | 33  (12%) | 33  (13%) | **0.01** | 0.01 |
| **Hospital admissions, previous year** |  |  |  |  |  |  |  |  |  |  |
| **Cardiovasc-ular cause** | 1341 (13%) | 474 (11%) | 333 (14%) | **0.07** | 0.07 | 31  (12%) | 32  (12%) | 30  (11%) | **0.01** | 0.01 |
| **Type 2 diabetes related cause** | 1456 (14%) | 548 (12%) | 343 (15%) | **0.04** | 0.04 | 35  (13%) | 35  (13%) | 32  (12%) | **0.01** | 0.01 |
| **Other cause** | 1947 (18%) | 751 (17%) | 439 (19%) | **0.03** | 0.03 | 46  (17%) | 45  (17%) | 43  (16%) | **0.02** | 0.03 |
| **Outpatient utilization, previous year** |  |  |  |  |  |  |  |  |  |  |
| **Cardiovasc-ular cause** | 1672 (16%) | 894 (20%) | 423 (18%) | **0.08** | 0.08 | 44  (17%) | 44  (16%) | 41  (16%) | **0.01** | 0.01 |
| **Type 2 diabetes cause** | 2571 (24%) | 1640 (37%) | 684 (29%) | **0.18** | 0.18 | 84  (32%) | 75  (28%) | 77  (30%) | **0.05** | 0.05 |
| **Other cause** | 7148 (67%) | 3357 (75%) | 1768 (75%) | **0.12** | 0.12 | 198 (75%) | 196 (74%) | 195 (74%) | **0.02** | 0.02 |
| **Total medications** |  |  |  | **0.08** | 0.08 |  |  |  | **0.06** | 0.06 |
| **1 (0-5)** | 4645 (44%) | 1814 (41%) | 1063 (45%) |  |  | 123 (47%) | 118 (45%) | 122 (47%) |  |  |
| **2 (6-10)** | 4815 (45%) | 2042 (46%) | 1013 (43%) |  |  | 111 (42%) | 119 (45%) | 110 (42%) |  |  |
| **3 (11-15)** | 994 (9%) | 523 (12%) | 246 (10%) |  |  | 25  (9%) | 25  (10%) | 26  (10%) |  |  |
| **4 (>15)** | 151 (1%) | 69 (2%) | 32  (1%) |  |  | 5  (2%) | 3  (1%) | 4  (1%) |  |  |

Additional baseline characteristics before and after propensity score using overlap weighting incl. standardized mean difference (SMD). The fifth and tenth columns contain the SMDs when DPP-4i is the reference, the sixth and eleventh columns contain the SMDs when GLP1-RA is the reference. Continuous variables are presented as median (interquartile range), whereas categorical variables are presented as n (%).
DPP-4i, dipeptidyl peptidase-4 inhibitor; GLP1-RA/GLP1, glucagon-like peptide-1 receptor agonist; SGLT2i, sodium-glucose cotransporter-2 inhibitor; COPD, chronic obstructive pulmonary disease; Renin-angiotensin inhibitors, angiotensin-converting enzyme (ACE) inhibitors and angiotensin receptor blockers (ARBs); NSAID, nonsteroidal anti-inflammatory drug; PPI, proton pump inhibitor; DM, diabetes mellitus.

**Figure S1.** Selection of study population flowchart.


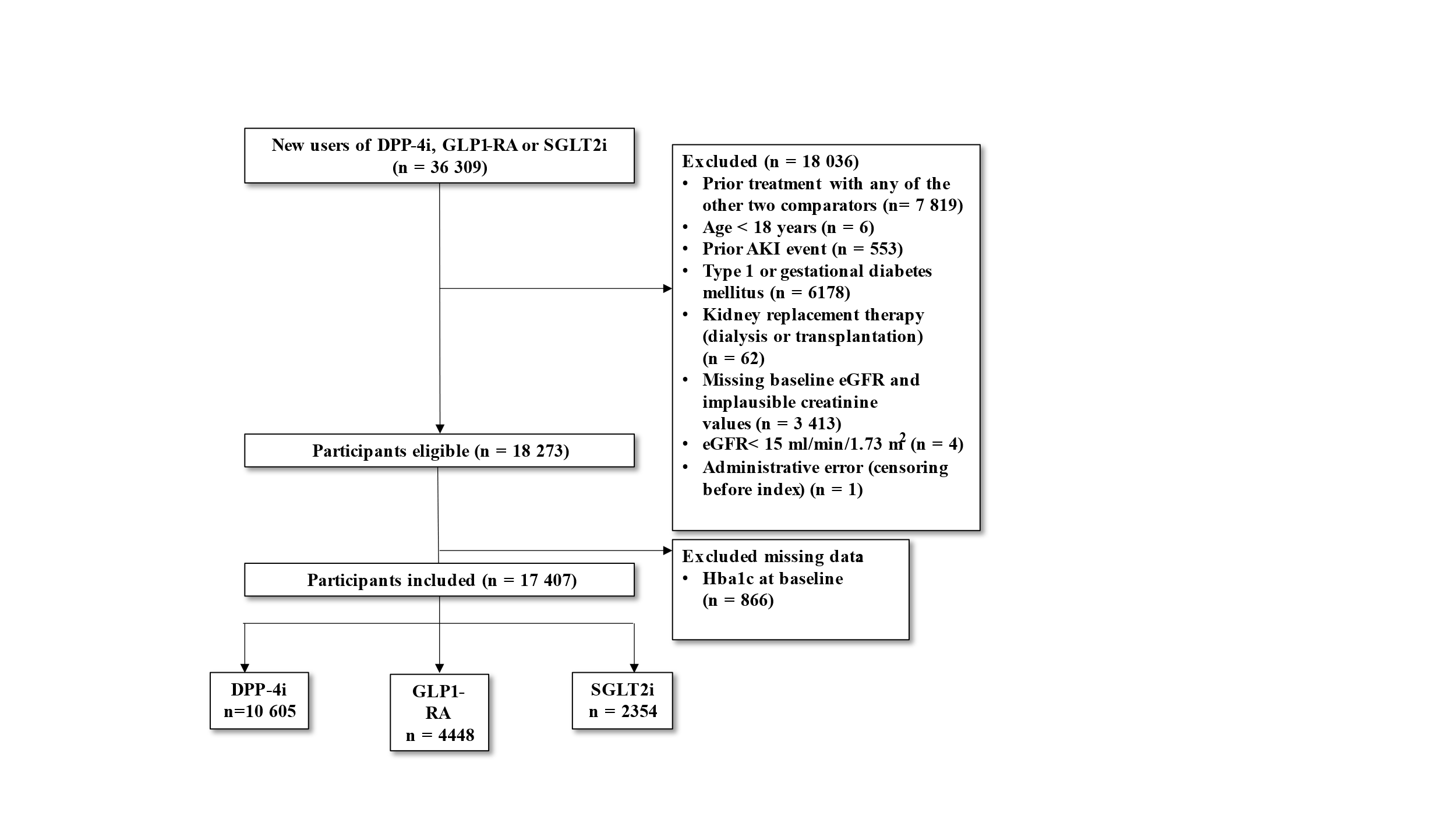


AKI, acute kidney injury; eGFR, estimated glomerular filtration rate; DPP-4i, dipeptidyl peptidase-4 inhibitor; GLP1-RA, glucagon-like peptide-1 receptor agonist; SGLT2i, sodium-glucose cotransporter-2 inhibitor.

**Figure S2.** Study design diagram.

**Follow-up window (outcomes)**

**[CED, censoring]**

**Cohort Entry Date [CED]**

**Study time**

**Cov: Labs (average)**

**[-183, CED]**

**Exc: Washout for exposure**

**[-365, CED]**

**Exc: Previous AKI, Type 1 or gestational diabetes, previous KRT**

**[∞, CED]**

Allowed calendar date range for cohort entry

[2008-01-01, 2018-12-31]

Dataset calendar period coverage

[2008-01-01, 2018-12-31]

**Exc: Age <18 years**

**[CED]**

**Exc: eGFR <15, missing eGFR in previous 12 months**

**[CED]**

**Cov: Demographics (age, sex, calendar year, education)**

**[CED]**

**Cov: Labs (average)**

**[-365, CED]**

**Cov: Healthcare utilization**

**[-365, CED]**

**Cov: Comorbidities**

**[∞, CED]**

**Cov: Medication use**

**[-183, CED]**

This figure describes the various time windows used to define exclusion criteria (“Exc”), covariates (“Cov”) and outcomes. Boxes are indicative of lengths but not to scale. Figure adopted from Schneeweiss S et.al. Graphical depiction of longitudinal study designs in health care databases. Ann Intern Med 2019;170:398-406.
AKI, acute kidney injury; eGFR, estimated glomerular filtration rate; KRT, kidney replacement therapy.
